# Circulating MiRNA-122 Levels Are Associated with Hepatic Necroinflammation and Portal Hypertension in HIV/HCV Coinfection

**DOI:** 10.1371/journal.pone.0116768

**Published:** 2015-02-03

**Authors:** Christian Jansen, Thomas Reiberger, Jia Huang, Hannah Eischeid, Robert Schierwagen, Mattias Mandorfer, Evrim Anadol, Philipp Schwabl, Carolynne Schwarze-Zander, Ute Warnecke-Eberz, Christian P. Strassburg, Jürgen K. Rockstroh, Markus Peck-Radosavljevic, Margarete Odenthal, Jonel Trebicka

**Affiliations:** 1 Department of Internal Medicine I, University of Bonn, Bonn, Germany; 2 Department of Internal Medicine III, Division of Gastroenterology & Hepatology, Vienna Hepatic Hemodynamic Lab, Medical University of Vienna, Vienna, Austria; 3 Department of Pathology, University of Cologne, Cologne, Germany; 4 Department of General, Visceral and Cancer Surgery, University of Cologne, Cologne, Germany; 5 German Center for Infection Research (DZIF), Partner Site Bonn-Cologne, Bonn, Germany; University of Navarra School of Medicine and Center for Applied Medical Research (CIMA), SPAIN

## Abstract

**Background:**

Introduction of combined antiretroviral therapy (cART) has improved survival of HIV infected individuals, while the relative contribution of liver-related mortality increased. Especially in HIV/HCV-coinfected patients hepatic fibrosis and portal hypertension represent the main causes of liver-related morbidity and mortality. Circulating miRNA-122 levels are elevated in HIV patients and have been shown to correlate with severity of liver injury. However, the association of miRNA-122 levels and hepatic fibrosis and portal hypertension remains to be explored in HIV/HCV coinfection.

**Methods:**

From a total of 74 (31% female) patients with HIV/HCV coinfection were included. Serum levels of miRNA-122 were analyzed by quantitative polymerase chain reaction (PCR) and normalized to SV-40 spike-in RNA. Hepatic venous pressure gradient (HVPG) was measured in 52 (70%) patients and the fibrosis stage was determined in 63 (85%) patients using transient elastography.

**Results:**

The levels of circulating miRNA-122 were increased in HIV/HCV coinfected patients and significantly correlated with the alanine aminotransferase (ALT) (r_s_ = 0.438; p<0.001) and aspartate transaminase AST values (r_s_ = 0.336; p = 0.003), but not with fibrosis stage (p = n.s.). Interestingly, miRNA-122 levels showed an inverse correlation with hepatic venous pressure gradient (HVPG) (r_s_ = −0.302; p = 0.03).

**Conclusion:**

Elevated miRNA-122 levels are associated with liver injury, and with low HVPG. Though, miRNA-122 levels are not suitable to predict the degree of fibrosis, they might function as indicators for portal hypertension in HIV/HCV coinfected patients.

## Introduction

HIV-infection is an emerging health problem worldwide. While overall survival of HIV-positive individuals has largely improved as a result of effective combined antiretroviral therapy (cART) [1–3], HIV patients with chronic hepatitis C virus (HCV) coinfection remain at increased risk of mortality [4]. Thus, HCV coinfection represents a leading cause of morbidity and mortality among HIV-patients, especially among men having sex with men [5] and intravenous drug users [6]. In addition, HCV-related liverdisease increases the hepatotoxicity of various drugs used for cART, while decreasing its efficacy [7]. On the other hand, HIV coinfection accelerates HCV-related fibrosis progression [8] and increases the risk for developing end-stage liver disease (ESLD) [9] with portal hypertension and hepatocellular carcinoma (HCC) [10]. Furthermore, HCV infection often remains untreated in HIV-positive patients due to contraindications, drug-drug interactions, and low response rates [11,12]. Therefore, the vast majority of HIV/HCV coinfected patients traditionally progressed to cirrhosis and developed liver-related complications [13,14]. Even though novel HCV therapies are now available [15], they are costly and even after sustained virological response (SVR), patients with advanced fibrosis remain at risk for ESLD and HCC [16–18]. Consequently, the progression of fibrosis and development of portal hypertension are strongest predictors of overall and liver-related mortality in HIV/HCV coinfected patients and risk factors for development of HCC [19–21]. The accurate and easy diagnosis of fibrosis and portal hypertension is an essential component for the management of HIV/HCV coinfected patients. To date the diagnosis of liver fibrosis and portal hypertension mainly relies on invasive methods, such as liver biopsy and measurement of the hepatic-venous pressure gradient (HVPG) [22,23], or on costly and limited devices (transient elastography, MRI-elastography, ARFI, shear-wave-elastography) [24–26]. Therefore, development of novel and simpler methods represent an unmet clinical need.

Previous studies on HIV and HCV patients could show that patterns of circulating miRNAs might mirror fibrosis stage and liver injury in patients with HIV and chronic HCV infection [27,28]. miRNA-122 levels are significantly up-regulated in HCV-infected patients [28] and higher levels of miRNA are possibly associated with improved long-term survival in cirrhotic patients with severe portal hypertension receiving TIPS [29]. miRNA levels correlate with the extent of liver injury [30–34] and especially miRNA-122 levels have been suggested as a biomarker in HIV/HCV coinfection reflecting the extent of liver injury [27,28]. Interestingly, while in acute HCV infection miRNA-122 reflects liver damage, in chronic HCV infection with established liver fibrosis the circulating levels of miRNA-122 were higher in mild fibrosis than in advanced stages [28]. Furthermore, serum levels of miR-122 were significantly higher in NAFLD patients than in healthy controls and serum levels of miR-122 correlated with inflammation activity in these patients [35].

This study aimed to assess the association of circulating levels of miRNA-122 with (i) liver injury and hepatic fibrosis and (ii) portal hypertension in the special population of HIV/HCV coinfected patients.

## Patients and Methods

### Patients and data collection

**Figure 1:**
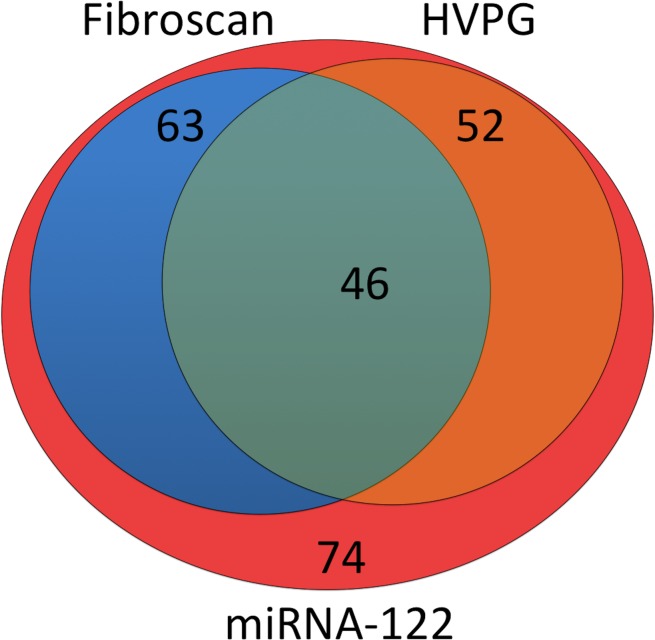
The Venn-diagram depicts the number of patients receiving HVPG-measurements and fibroscan. Circulating miRNA-122 levels were measured in seventy-four patients with HIV/HCV coinfection. Hepatic venous pressure gradient (HVPG) was measured in fifty-two patients. The fibrosis stage was determined using transient elastography in 63 patients.

We retrospectively included seventy-four (31% female) patients with HIV/HCV coinfection. The median age was thirty-seven years with a range from nineteen to sixty-three. Hepatic venous pressure gradient (HVPG) was measured in fifty-two (70%) patients. The fibrosis stage was determined using transient elastography (Fibroscan, Echosens, France) in 63 patients (85%). The Venn-diagramm (Fig. 1) highlights the distribution of available HVPG and liver stiffness data in our patient cohort. Circulating miRNA-122 levels were measured in peripheral blood collected during routine blood sampling in all patients. Furthermore, biochemical parameters were analyzed using standard methods.

The HCV-genotype (GT) distribution was as follows: GT1 n = 43 (58%), GT2 n = 3 (4%), GT3 n = 15 (20%), GT4 n = 12 (16%), GT6 n = 1 (1%). None of patients received HCV therapy at time of miRNA-122 measurement. Therafter 12 patients received further no therapy, while sixty-one were treated with interferon and ribavirin and only one received triple therapy with boceprevir, interferon and ribavirin.

57 patients were on cART and all received a nucleos(t)idic reverse transcriptase inhibitor (NRTI) as a backbone of their cART (mostly tenofovir/emtricitabine, or abacavir/lamivudine). We then stratified the cART patients into 3 groups: 34 patients were combined with protease inhibitors (PIs: Lopinavir, Atazanavir, and ritonavir booster), 19 patients were combined with non-nucleosidic reverse trancriptase inhibitors (NNRTIs, mostly efavirenz), and 4 patients were combined with the integrase inhibitor (II) raltegravir.

The patients gave their written consent for all procedures and the local ethics committee of the University of Bonn (Nr. 069/10) as well as the local ethics committee of the University of Vienna (EK 005/2005) approved the study in accordance with the Declaration of Helsinki.

### Measurement of portal pressure by hepatic venous pressure gradient and liver stiffness by transient elastography

HVPG measurements were performed as previously described [22,29,36,37]. Under local anesthesia and ultrasound guidance, the right internal jugular vein was cannulated using the Seldinger technique. Under x-ray guidance, a balloon catheter was placed in a major hepatic vein. To calculate HVPG, three repeated measurements of free and wedged hepatic venous pressure were performed. Pressure curves were continuously recorded using a licensed software (S5 Collect, Vienna, Austria). Transient elastography (FibroScan, Echosens, France) was used for measurement liver stiffness in sixty-three patients as previously described [22,29,36,37].

### miRNA isolation and quantification by real-time PCR

Peripheral blood samples were collected from patients as part of the routine investigation. Blood samples were centrifuged at 3000 rpm for 15 minutes at 4°C and sera were stored at −80°C. RNA was isolated from serum samples using the Qiazol reagent following the instructions of the supplier (Qiagen, Hilden, Germany) as previously described [27,28]. SV40-miRNA (Qiagen) was added to serum samples (2 pmol/200 μl) prior to the RNA isolation procedure for later normalization of circulating miRNA-122

miRNA was analyzed by a two-step real-time PCR using the miScript-Reverse Transcription Kit and the miRNA-SYBR Green PCR Kit (Qiagen, Hilden, Germany). miRNA-122 and SV-40 primers used for real-time PCR were selected and purchased from the GeneGlobe Search Center (Qiagen, Hilden, Germany). All steps were performed in triplicate and in agreement with the supplier’s guidelines. For normalization of extracellular miRNA-122 levels, spike-in SV40-miRNA (Qiagen, Hilden, Germany) was used.

### Statistical analysis

Mann-Whitney test was used for comparisons between groups. Differences between more than two groups were analyzed using Kruskal-Wallis-Test. The correlations were analyzed using Spearman correlation coefficient. Analysis of the area the receiver operating characteristics curve (AUROC) was used to determine a potential cut-off-value to predict portal hypertension. All statistical analyses were performed using SPSS 22 for Windows (SPSS Inc. Chicago, IL, USA).

## Results

### Clinical and hemodynamic characteristics of patients

**Figure 2:**
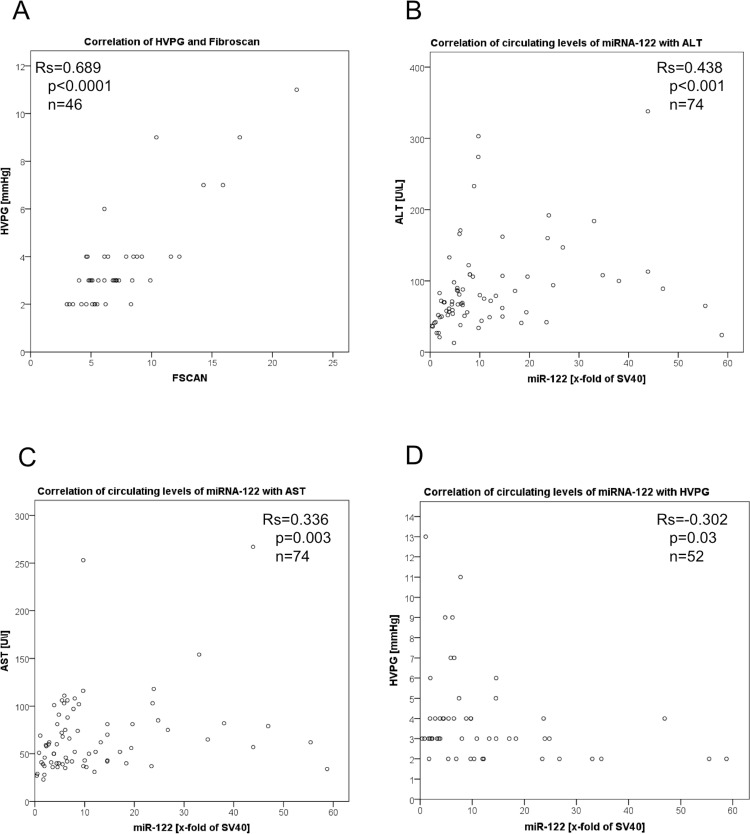
Serum levels of circulating miRNA-122 correlation with ALT-levels and HVPG in HIV/HCV co-infected patients, as well as HVPG with FibroScan. HVPG correlates (r_s_ = 0.689; p = 1.47*10^−7^) with transient elastography assessed by FibroScan© (A). The levels of circulating miRNA-122 measured in peripheral blood showed significant correlations with ALT (r_s_ = 0.438; p<0.001) (B) and AST (r_s_ = 0.336; p = 0.003) (C). The levels of circulating miRNA-122 measured in peripheral blood showed significant (r_s_ = 0.−302; p = 0.03) inverse correlation with HVPG (D). Data were presented using Spearman coefficient r_s_ and p-values. The levels of miRNA-122 were normalized to SV40 and shown as the x-fold of SV40.

The clinical and hemodynamic characteristics of the investigated patients are listed in Table 1. The median age of these patients was 37 years with a range of 19 to 63 years and 31% were female. Median liver stiffness evaluated using transient elastography was 6.2 kPa with a range 3–22 kPa. Thirty-six patients showed no or F1 fibrosis, twenty-one patients showed stage F2 and F3 and six patients presented with cirrhosis (F4). HVPG was measured in fifty-two patients. The median was 3 mmHg with a range between 2–13 mmHg. HVPG correlated with liver stiffness (r_s_ = 0.689; p = 1.17*10^−7^; Fig. 2A).

**Table 1:** Clinical parameters of patients.

Parameters	Number of patients
Gender [female/male]	23/51
Age [years]	37 (19–63)
Body weight [kg]	70 (45–117)
Fibrosis stage according to liver stiffness (F0–F1/ F2–F3/F4)	36/21/6
HVPG (0–4/5–10/>10mmHg)	42/8/2
cART regimen (n = 57) combined with NRTI	+PI (n = 34), +NNRTI (n = 19), +II (n = 4)

Data are shown as median and range; Liver stiffness as measured by transient elastography was converted into the respective fibrosisstage (F0–F1<7kPa;F2–F3: 7kPa-12.5kPa; F4>12.5kPa); HVPG, hepatic venous pressure gradient; cART, combined antiretroviral therapy; NRTI, nucleos(t)idic reverse transcriptase inhibitor; NNRTI, non-nucleos(t)idic reverse transcriptase inhibitor; PI, protease inhibitor; II, integrase inhibitor.

### Biochemical characteristics of patients

Most patients showed elevated levels of aminotransferases indicating liver injury with median levels of alanine aminotransferase (ALT) at 70 U/L and of aspartate transaminase (AST) at 58.5 U/L. Median HCV-RNA levels were 1.6 * 10^6^ IU/mL. In contrast, cholinesterase levels, as well as gamma-glutamyl transferase (γGT), alkaline phosphatase (AP), albumin and platelet count were largely within normalranges (Table 2).

**Table 2:** Laboratory parameters of patients.

Parameters	Value	n
HVPG (mmHg)	3 (2–13)	52
ALT (U/L)	70 (13–338)	74
AST (U/L)	58.5 (23–267)	74
CHE (U/mL)	7.4 (3–13)	72
γGT (U/L)	84.5 (14–414)	74
AP (U/L)	92.5 (46–270)	72
Albumin (U/L)	41.8 (32.8–52.7)	74
Platelet count(10^9^/L)	186 (47–330)	74
HCV RNA (IU/mL)	1.6 10^6^ (2280–37.7 10^6^)	74
CD4+ T cell count (/μL)	526 (16–1541)	73
miRNA-122	6.8 (0.4–58.8)	74

Data are shown as median and range; HVPG, hepatic venous pressure gradient; ALT, alanine aminotransferase; AST, aspartate transaminase; CHE, cholinesterase; γGT, gamma-glutamyl transferase; AP, alkaline phosphatase; HCV-RNA, hepatitis-C-virus ribonucleic acid; CD4, CD4+ T; helper cells; miRNA-122, micro ribonucleic acid 122 displayed as x- fold of SV40.

### Levels of circulating miRNA-122 reflect hepatic injury

Interestingly levels of circulating miRNA-122 showed significant correlations with ALT (r_s_ = 0.438; p<0.001; Fig. 2B) as well as AST (r_s_ = 0.336; p = 0.003; Fig. 2C) and thus with hepatic inflammation.

However, there are no significant correlations with miRNA-122 levels and γGT (r_s_ = −0.015; p = 0.898), CHE (r_s_ = 0.086; p = 0.475), HCV viral load (R_s_ = 0.001; p = 0.994), AP (r_s_ = −0.124; p = 0.298), albumin (r_s_ = −0.027; p = 0.821) or platelet count (r_s_ = 0.037; p = 0.753). In respect to the miRNA-122 levels, no significant differences could be observed if patients were stratified by sex (p = 0.528), as well as in regarded to their HIV-treatment (p = 0.565) or HCV genotype (p = 0.918)

### Levels of circulating miRNA-122 reflect portal hypertension

Interestingly, the miRNA-122 levels showed no correlation with liver stiffness (r_s_ = 0.041; p = 0.751). However, the levels of circulating miRNA-122 showed a significant inverse correlation with HVPG (r_s_ = −0.302; p = 0.03; Fig. 2D).

**Figure 3:**
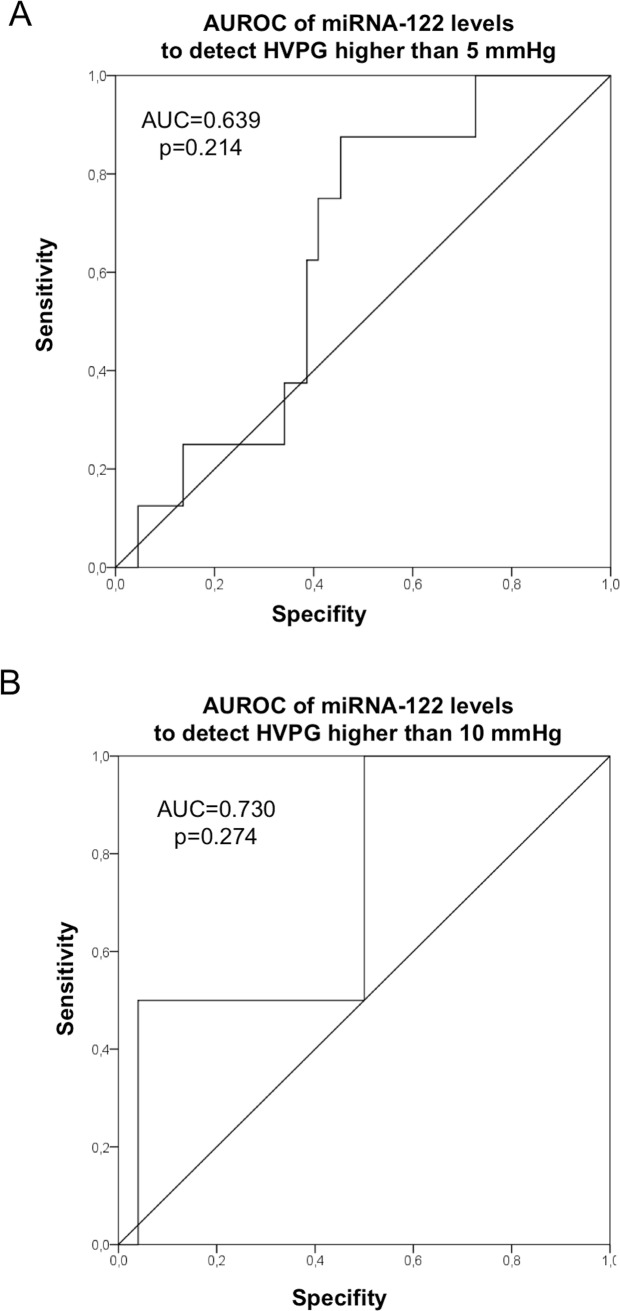
miRNA-122 could not predict portal hypertension. AUROC analysis demonstrated that we unfortunatelydid not succeeded to define a cut-off value to predict portal hypertension. (A) AUC of 0.639 (p = 0.214) for detecting an HVPG of 5mmHG. (B) AUC of 0.73 (p = 0.274) for detecting an HVPG of 10mmHg—indicating clinically significant portal hypertension.

AUROC analysis for circulating miRNA-122 to identify patients with HVPG 5mmHg (indicating elevated portal pressure) or above 10 mmHg (clinically significant portal hypertension) showed no significant results. For both miRNA-122 cut-of-values could not be determined (for 5 mmHG AUC: 0.639, p = 0.214; for 10mmHg AUC: 0.730, p = 0.274; Fig. 3A, 3B).

## Discussion

The current study suggests, that miRNA-122 levels are associated with liver injury but inversely correlate with portal pressure in patients with HIV/HCV coinfection. Various microRNAs detectable in tissue and body fluids are now being evaluated as biomarker for multiple diseases. In particular, a role of miRNA-122 as the most abundant miRNA in the liver has been discussed as a surrogate marker for liver injury and fibrosis development [27,28].

The progression of liver fibrosis and development of ESLD in patients with HIV/HCV coinfection is accelerated compared to HCV mono-infected individuals [8,13,14]. Progressive fibrosis goes along with gradual increases in portal pressure and may finally progress to cirrhosis with portal hypertension and associated complications such as variceal bleeding or ascites. The most accurate method to quantify portal pressure is the invasive measurement of the HVPG. Apart from the logistical effort and the technical requirements needed, the patients’ tolerance is often low due to invasive nature and discomfort related to the technique [38]. Thereby, this method might be unfavorable as primary screening technique in risk groups and non-invasive tests for early detection of liver fibrosis and portal hypertension are needed.

Serum biomarker should offer a non-invasive alternative for assessment of fibrosis and portal hypertension in chronic liver disease. In this study we analyzed miRNA-122 in peripheral blood of seventy-four serum samples of HIV/HCV coinfected patients. Interestingly levels of circulating miRNA-122 showed significant correlations with ALT indicating that increased miRNA-122 levels might reflect ongoing hepatocellular damage and hepatic necroinflammation. This confirms the results from our previous studies of miRNA-122 levels in patients with HCV mono-infection [28]. We further could confirm our recent observation in HIV patients, since miRNA-122 was increased in HIV patients with HCV-coinfection [27,28]. Interestingly, lower miRNA-122 levels were associated with higher hepatic venous pressure gradients in HIV/HCV coinfected patients. These observations are in line with our previous data showing that the levels of miRNA-122 decrease with more advanced liver fibrosis stages [28]. Therefore this study provides important evidence that the release of miRNA-122 is a response to injury and associated with necroinflammation of hepatocytes. However with decreasing functional hepatocyte mass, which occurs in progressive fibrosis and in advanced cirrhosis with portal hypertension, miRNA-122 do not hold their initially very high levels due to the injury, but show a gradual decrease correlating inversely with fibrosis and portal pressure. Thus, high inflammatory activity with ongoing hepatocellular damage of any etiology might be associated with high expulse of miRNA-122 into the circulation, probably regardless of etiology [28] [35].

Unfortunately, we did not succeeded to define a clinically useful miRNA-122 cut-off value to predict portal hypertension by AUROC analysis. This might be due to the fact that most of our patients showed normal HVPG values, and the overall range of HVPG values was limited.

This study describes a well characterized patients cohort, including laboratory and clinical data, as well as invasive measurements of the portal pressure (HVPG) and Fibroscan assessment. Despite these strengths, the present study has several limitations. The sample size is rather small and not all patients had simultaneous HVPG and transient elastography measurement. Additionally, no liver biopsies were obtained, as the golden standard for fibrosis and inflammation.

Besides miRNA-122, further research on non-invasive markers has been published as the enhanced liver fibrosis panel [39], von Willebrand factor [40], circulating marker of collagen type III, type IV and V [41]. However, more research is still needed to indentify and validate these or other novel non-invasive markers.

In summary, circulating miRNA-122 levels in HIV/HCV coinfection are inversely correlated with portal pressure, but directly correlated with serum AST and ALT levels indicating hepatic inflammation. Thus, miRNA-122 levels might be useful surrogate markers of ongoing hepatic necroinflammation in HIV/HCV coinfection. However—despite an indirect correlation of miRNA-122 and portal pressure was noted—miRNA-122 levels might not be a suitable marker to accurately predict the stage of fibrosis or the degree of portal hypertension in HIV/HCV coinfected patients.
